# Bile aspiration technique for successful guidewire manipulation in endoscopic ultrasound-guided hepaticogastrostomy with antegrade stenting

**DOI:** 10.1055/a-2155-5496

**Published:** 2023-09-15

**Authors:** Yuichi Takano, Masataka Yamawaki, Jun Noda, Tetsushi Azami, Fumitaka Niiya, Fumiya Nishimoto, Masatsugu Nagahama

**Affiliations:** Division of Gastroenterology, Department of Internal Medicine, Showa University Fujigaoka Hospital, Yokohama, Kanagawa, Japan

The greatest technical challenge in endoscopic ultrasound (EUS)-guided hepaticogastrostomy with antegrade stenting is breaching the bile duct stricture with guidewire 1 2 . Herein, we report a case in which a novel bile aspiration technique achieved smooth guidewire breaching of the biliary stricture.

A woman in her 70 s was admitted to our hospital for obstructive jaundice caused by unresectable pancreatic head cancer. Transpapillary biliary cannulation was unsuccessful due to duodenal invasion of the tumor, and the patient underwent EUS-guided hepaticogastrostomy with antegrade stenting. An echoendoscope (GF-UCT260; Olympus, Tokyo, Japan) was inserted and the intrahepatic bile duct (B3) was punctured with a 19-gauge needle (EZshot3, Olympus). After cholangiography, a 0.025-inch guidewire (Visiglide2, Olympus) was inserted, followed by a tapered-tip catheter (Shoren, KANEKA, Osaka, Japan). Severe stenosis was observed in the distal bile duct. A 0.025-inch hydrophilic guidewire (Radifocus; TERUMO, Tokyo, Japan) was used to attempt breaching the stenosis; however, the guidewire could not be visualized due to retention of the contrast medium ( Fig. 1 ). A total of 26 mL bile was removed using the catheter and the guidewire was visualized after successful aspiration. Moreover, EUS confirmed the collapse of the intrahepatic bile duct. The stricture was successfully breached, and the guidewire was placed in the duodenum ( Fig. 2 ). An uncovered self-expandable metallic stent (diameter, 8 mm; length, 80 mm; YABUSAME Neo, KANEKA) was placed in the distal bile duct. Finally, a 7-Fr plastic stent (Through & Pass TYPE IT; Gadelius Medical, Tokyo, Japan), 14 cm in length, was deployed as the hepaticogastrostomy and the procedure was completed without any procedural adverse events ( Video 1 ). Consequently, her jaundice rapidly improved and chemotherapy was initiated.

**Fig. 1:**
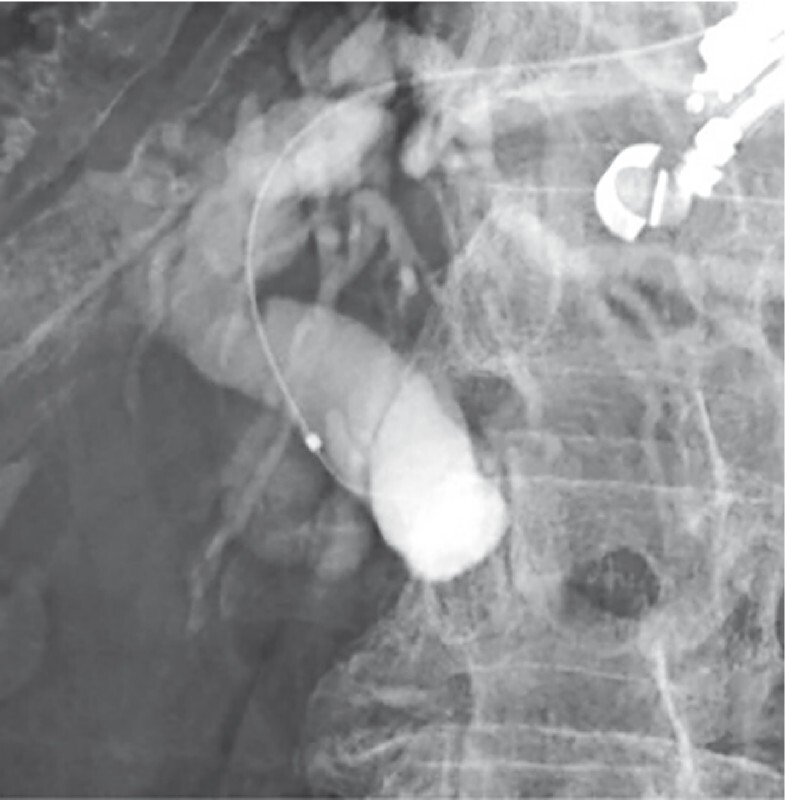
Severe stenosis was observed in the distal bile duct on cholangiography. A 0.025-inch hydrophilic guidewire was used to attempt breaching the stenosis; however, the guidewire could not be visualized due to retention of the contrast medium.

**Fig. 2:**
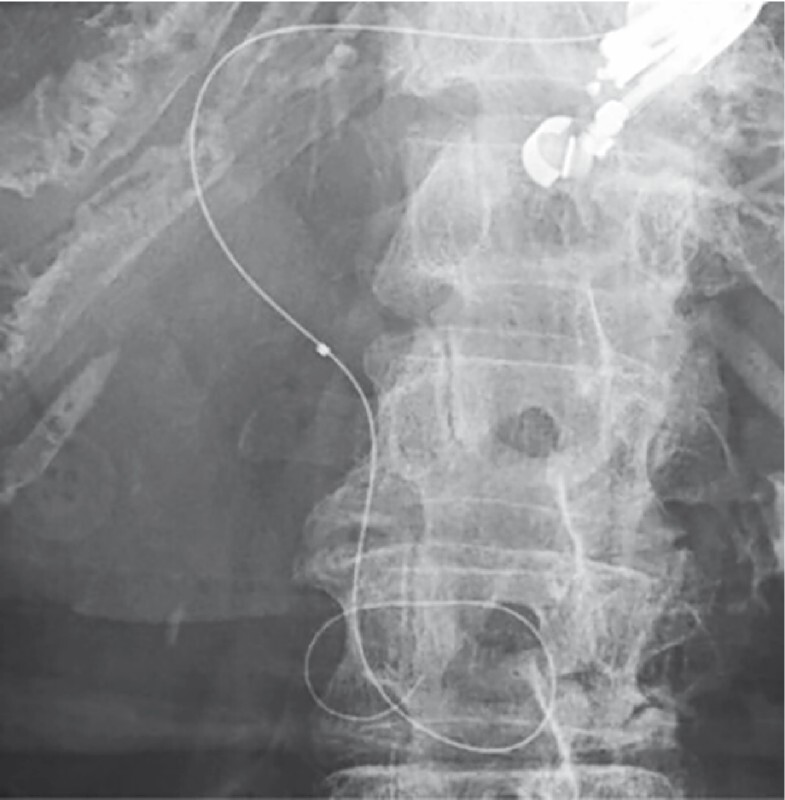
A total of 26 mL of bile was removed using the catheter. The contrast medium completely disappeared. The guidewire could be visualized after successful aspiration. The stricture was successfully breached, and the guidewire was placed in the duodenum.

**Video 1** Bile aspiration technique in endoscopic ultrasound-guided hepaticogastrostomy with antegrade stenting.


The advantages of the bile aspiration technique include improved guidewire visibility and reduced bile duct diameter, which facilitate the stenosis breach. It is a simple and effective method in EUS-guided hepaticogastrostomy with antegrade stenting.

Endoscopy_UCTN_Code_TTT_1AS_2AD

## Notes

Endoscopy E-Videos

https://eref.thieme.de/e-videos
*E-Videos* is an open access online section of the journal *Endoscopy* , reporting on interesting cases and new techniques in gastroenterological endoscopy. All papers include a high-quality video and are published with a Creative Commons CC-BY license. Endoscopy E-Videos qualify for HINARI discounts and waivers and eligibility is automatically checked during the submission process. We grant 100% waivers to articles whose corresponding authors are based in Group A countries and 50% waivers to those who are based in Group B countries as classified by Research4Life (see: https://www.research4life.org/access/eligibility/ ). This section has its own submission website at https://mc.manuscriptcentral.com/e-videos
